# Nighttime intensive care unit discharge and outcomes: A propensity matched retrospective cohort study

**DOI:** 10.1371/journal.pone.0207268

**Published:** 2018-12-13

**Authors:** Thiago Domingos Corrêa, Carolina Rodrigues Ponzoni, Roberto Rabello Filho, Ary Serpa Neto, Renato Carneiro de Freitas Chaves, Andreia Pardini, Murillo Santucci Cesar Assunção, Guilherme De Paula Pinto Schettino, Danilo Teixeira Noritomi

**Affiliations:** 1 Dept. of Critical Care Medicine, Hospital Israelita Albert Einstein, São Paulo, Brazil; 2 Dept. of Critical Care Medicine, Hospital Municipal Moysés Deutsch, São Paulo, Brazil; 3 Dept. of Intensive Care, Academic Medical Center, Amsterdam, The Netherlands; University of Notre Dame Australia, AUSTRALIA

## Abstract

**Background:**

Nighttime ICU discharge, i.e., discharge from the ICU during the night hours, has been associated with increased readmission rates, hospital length of stay (LOS) and in-hospital mortality. We sought to determine the frequency of nighttime ICU discharge and identify whether nighttime ICU discharge is associated with worse outcomes in a private adult ICU located in Brazil.

**Methods:**

Post hoc analysis of a cohort study addressing the effect of ICU readmissions on outcomes. This retrospective, single center, propensity matched cohort study was conducted in a medical-surgical ICU located in a private tertiary care hospital in São Paulo, Brazil. Based on time of transfer, patients were categorized into nighttime (7:00 pm to 6:59 am) and daytime (7:00 am to 6:59 pm) ICU discharge and were propensity-score matched at a 1:2 ratio. The primary outcome of interest was in–hospital mortality.

**Results:**

Among 4,313 eligible patients admitted to the ICU between June 2013 and May 2015, 1,934 patients were matched at 1:2 ratio [649 (33.6%) nighttime and 1,285 (66.4%) daytime discharged patients]. The median (IQR) cohort age was 66 (51–79) years and SAPS III score was 43 (33–55). In-hospital mortality was 6.5% (42/649) in nighttime compared to 5.6% (72/1,285) in daytime discharged patients (OR, 1.17; 95% CI, 0.79 to 1.73; p = 0.444). While frequency of ICU readmission (OR, 0.95; 95% CI, 0.78 to 1.29; p = 0.741) and length of hospital stay did not differ between the groups, length of ICU stay was lower in nighttime compared to daytime ICU discharged patients [1 (1–3) days vs. 2 (1–3) days, respectively, p = 0.047].

**Conclusion:**

In this propensity-matched retrospective cohort study, time of ICU discharge did not affect in-hospital mortality.

## Background

Intensive care unit (ICU) beds represent an expensive and scarce resource in health care systems worldwide [1]. The demand for ICU beds has supplanted bed availability in many low [2] and high-income countries [3, 4]. Limited ICU bed availability may contribute to premature ICU discharge and a non-ideal treatment transition program between the ICU and the ward [5]. Premature ICU discharge has been associated to an increased risk of unplanned ICU readmission and death [6]. Therefore, ICU discharge polices and critical care transition programs constitute important elements in the routine of hospital care [7–9].

Nighttime ICU discharge, i.e., discharge from ICU during the night hours, has been associated with increased readmission rates [10–13], hospital length of stay (LOS) [11] and in-hospital mortality [13–15]. The reasons for worse outcomes among patients discharged from the ICU during nighttime in comparison to daytime are multifactorial and not completely understood [9]. Patients’ severity of illness at ICU admission and discharge [16, 17], reduced staff levels [18], decreased nurse-to-patient ratio [19], poor surveillance after ICU discharge [20], destination after ICU discharge (ward vs. step-down unit) [21], pressure for ICU beds leading to premature ICU discharge of a patient to make an ICU bed available for a more acutely or severely ill patient [5, 22], and delayed ICU discharge often due to a lack of ward beds are all likely to contribute [23].

Yang and cols. demonstrated in a systematic review and meta-analysis of fourteen studies conducted in adult ICUs located in north America, Europe, Australia and New Zealand that nighttime ICU discharge was associated with a thirty percent increase in the risk of in-hospital mortality [14]. Similar findings were recently reported by Vollam and cols. in another meta-analysis including 1,191,178 patients from eighteen cohort studies [13]. Nevertheless, since most of the knowledge about epidemiology of time of ICU discharge and its impact on outcomes is restricted to developed countries, we sought to determine the frequency of nighttime ICU discharge and identify whether nighttime ICU discharge is associated with worse outcomes in a private adult ICU located in Brazil.

## Objective

Our objective was to evaluate the effect of time of ICU discharge on in-hospital mortality and ICU readmission rate in a tertiary care hospital. Secondary objective was to address the question of whether the location after ICU discharge (wards or step-down unit) affects outcomes in a pressure-free environment for ICU beds rationing and with full availability of step-down unit beds.

## Methods

The present study is a post hoc analysis of a retrospective single center cohort study that investigated the effect of ICU readmissions on resource use, in-hospital mortality and outcomes [15]. The original study and this post hoc analysis were approved by the Local Ethics Committee at Hospital Israelita Albert Einstein with waiver of informed consent (CAAE:54065716.3.0000.0071).

### Setting

This study was conducted in a private tertiary care hospital in São Paulo, Brazil, comprising 662 inpatient beds, two adult medical-surgical, open model ICUs with 44 beds in total and 91 step-down unit beds.

### Patients

**Fig 1:**
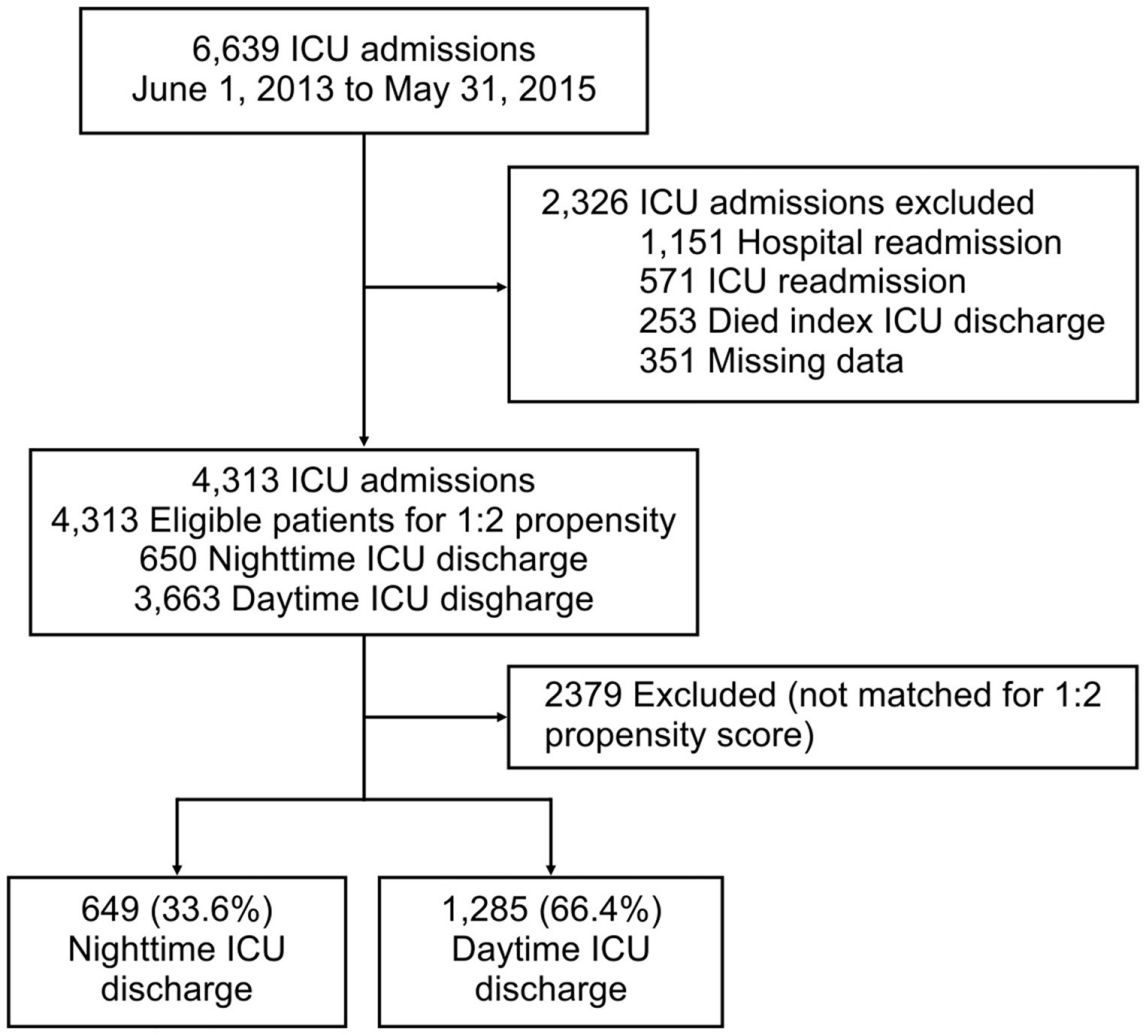
Patient flow charge.

We included all consecutive patients aged ≥18 years old admitted to the ICU and discharged alive between June 1, 2013 and May 31, 2015. Patients who died during the index ICU stay and those with missing core data [age, gender, time of ICU discharge, ICU admission diagnosis, Simplified Acute Physiology score (SAPS 3 score) at ICU admission [24] destination at ICU discharge, ICU and hospital LOS and vital status at hospital discharge], were excluded (Fig 1).

### Data collection and study variables

All study data were retrieved from Epimed Monitor System (Epimed Solutions, Rio de Janeiro, Brazil), which is an electronic structured case report form where patients data are prospectively entered by trained ICU case managers [25, 26].

Collected variables included demographics, comorbidities, location before ICU admission, reason for ICU admission, SAPS 3 score at ICU admission [24], time of ICU admission and discharge, ICU admission diagnosis, supportive therapy (need for vasopressors, mechanical ventilation, noninvasive mechanical ventilation and renal replacement therapy) at ICU admission and during index ICU stay, destination at ICU discharge, frequency of weekend ICU discharge, frequency of ICU readmission, ICU and hospital LOS and in-hospital mortality.

### Definitions

Based on the time of transfer, patients were categorized into daytime (7:00 am to 6:59 pm) and nighttime (7:00 pm to 6:59 am) ICU discharge [11]. Weekend ICU discharge was defined as any discharge from the ICU occurring between Saturday midnight and Sunday at 11:59 pm [27]. Readmission was defined as ICU admission of a patient who had been previously admitted to the ICU (index ICU admission) during the same hospitalization stay [15]. Only the first ICU readmission was included in this analysis. In case of hospital readmissions during the study period, only the first hospital admission was considered.

### ICU and step-down unit characteristics

On-duty ICU physicians are available 24 hours a day at a rate of one intensivist per every ten beds. There is no reduction in personnel or in ICU activities during night shifts or at weekends. Multidisciplinary clinical rounds involving ICU physicians, nurses, respiratory therapists, nutritionists, psychologist and clinical pharmacists are performed daily. ICU admissions are made by on-duty intensivists, whereas discharge is a consensus decision-making process involving on-duty intensivists and the physician who will accept the patient outside the ICU.

In the step-down unit, on-duty ICU physicians are available 24 hours a day at a rate of one intensivist per each 20 beds during day shifts and one intensivist per every 40 beds during night shifts. There is no reduction in non-physician multidisciplinary team during night shifts or at weekends. Multidisciplinary care involving nurses, respiratory therapists, nutritionists, psychologist and clinical pharmacists are available 24 hours a day.

Patients are considered eligible for ICU discharge to a step-down unit when they are hemodynamically, respiratory, metabolically and neurologically stable, and therefore no longer requiring intensive care, although still demanding more care than provided at the ward. The hospital has a 24-hour coverage by a rapid response team (RRT) led by ICU physicians who immediately respond to ward patients experiencing acute clinical deterioration.

### Outcomes

The primary outcome of interest was in-hospital mortality. Secondary outcomes included frequency of ICU readmission, number of ICU readmissions, and length of ICU and hospital stay.

### Statistical analysis

Categorical variables are presented as absolute and relative frequencies. Continuous variables are presented as median with interquartile ranges (IQR). Normality was assessed by the Kolmogorov-Smirnov test. Comparisons were made between nighttime and daytime ICU discharge patients. Categorical variables were compared with chi-square test. Continuous variables were compared using independent t test or Mann-Whitney U test in case of non-normal distribution. Primary outcome was assessed by unadjusted logistic regression analysis using daytime ICU discharge as a reference level and presented as odds ratio (OR) along with 95% confidence interval (95%CI).

Propensity scores for nighttime ICU discharge were estimated for each patient with logistic regression using nineteen clinically relevant patient characteristics [age, gender, SAPS III score, reason for index ICU admission, index admission source, presence of systemic hypertension, diabetes mellitus, cancer, congestive heart failure, chronic obstructive pulmonary disease, chronic kidney disease and liver cirrhosis, supportive therapy during the ICU stay (need for vasopressors, mechanical ventilation, noninvasive mechanical ventilation and renal replacement therapy), index weekend ICU discharge, ICU LOS and destination at index ICU discharge] [28]. Based on the propensity score weighted estimators we constructed a propensity score-matched cohort. Matching was performed using nearest neighbor matching without replacement, with each patient with a nighttime ICU discharge matched to two patients with daytime ICU discharge. A caliper width of 0.10 of the standard deviation of the logit of the propensity score was used for the development of matching [29, 30].

Statistical tests were two-sided. A p<0.05 was considered statistically significant. Statistical analyses were performed using IBM SPSS Statistics version 22.0 for Windows.

## Results

### Characteristics of studied population

**Fig 2:**
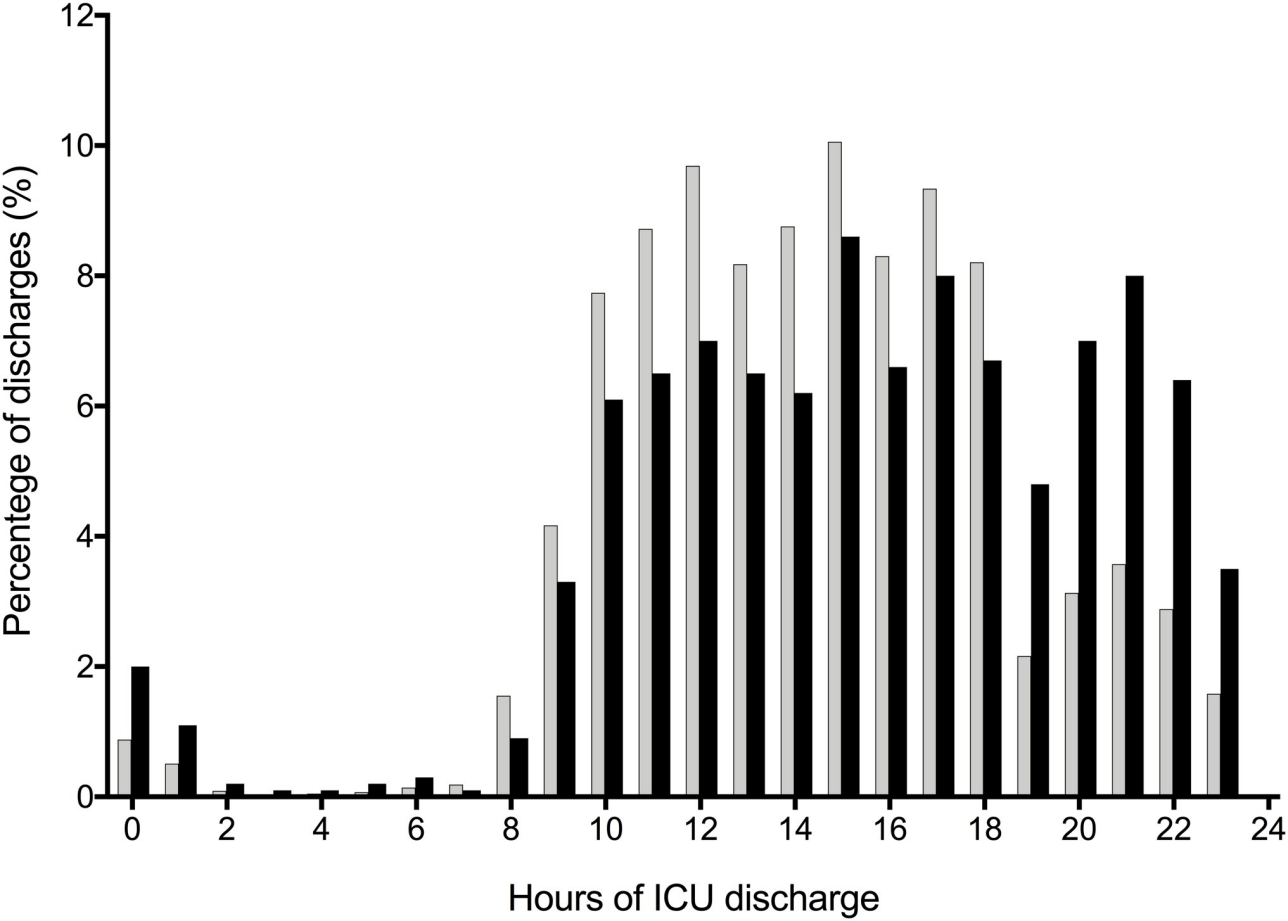
Histogram showing the distribution of hours of intensive care unit discharge before and after propensity score matching. Grey bars represent the cohort (n = 4,313 patients) before propensity score matching and black bars represent the cohort (n = 1,934 patients) after propensity score matching.

Between June 1, 2013 and May 31, 2015, a total of 6,639 patients were admitted to the ICU (Fig 1). Following exclusion of 2,326 ineligible ICU admissions, 4,313 patients [650 (15.1%) nighttime ICU discharge and 3,663 (84.9%) daytime ICU discharge] were eligible for propensity score matching, of which 1,934 patients were successfully matched (S1 and S2 Figs). Out of those, 649 (33.6%) patients were discharged from the ICU during nighttime and 1,285 (66.4%) patients were discharged during daytime (Fig 1). A histogram showing the distribution of hours of ICU discharge for the study population (n = 1,934 patients) is shown in Fig 2. The median (IQR) hospital and ICU bed occupancy rates during the study period were, respectively, 86.1% (83.7%–87.1%) and 85.4% (82.6%–89.7%).

### Cohort before propensity score matching

#### Baseline characteristics

Before propensity score matching, compared to daytime ICU discharged patients, nighttime discharged patients had a higher [median (IQR)] SAPS III score at index ICU admission [42 (33–55) vs. 40 (31–52), respectively for nighttime and daytime ICU discharge; p<0.001)], were more frequently admitted due to medical reasons [399/650 (61.4%) patients vs. 1,919/3,663 (52.4%) patients, respectively for nighttime and daytime ICU discharge; p<0.001] and were more frequently admitted to the ICU from the emergency department [265/650 (40.8%) patients vs. 1,278/3,663 (34.9%) patients, respectively for nighttime and daytime ICU discharge; p<0.001] (S1 Table).

Nighttime ICU discharged patients were more frequently discharged to a step-down unit [402/650 (61.8%) patients vs. 2,091/3,663 (57.1%) patients, respectively for nighttime and daytime ICU discharge; p<0.001] and less frequently discharged to the ward [206/650 (31.7%) patients vs. 1,412/3,663 (38.5%), respectively for nighttime and daytime ICU discharge; p<0.001]. Finally, the frequency of weekend ICU discharge was lower in nighttime compared to daytime ICU discharged patients [18.9% vs. 27.3%; respectively, p<0.001) (S1 Table).

#### Outcomes

Before matching, hospital mortality was 6.5% (42/650 patients) in nighttime discharged patients compared to 5.1% (187/3,663 patients) daytime discharged patients (OR, 1.28; 95% CI, 0.91 to 1.81; p = 0.156) (S2 Table). Frequency of ICU readmissions and length of ICU and hospital stay did not differ between nighttime and daytime ICU discharged patients (S2 Table).

### Cohort after propensity score matching

#### Baseline characteristics

The propensity-matched cohort had a median (IQR) age of 66 (52–79) years, 57.6% (1,114/1,934) of patients were men with a median (IQR) SAPS III score of 43 (33–55) (Table 1). The study groups were well balanced with respect to age, gender, SAPS III score at index ICU admission, reason for index ICU admission, admission source, prevalence of co-morbidities, admission diagnosis and the need of supportive therapy on index ICU admission (Table 1). Patients were more frequently discharged to a step-down unit [1,184/1,934 (61.2%) patients)] followed by the ward [629/1,934 (32.5%) patients], with no difference between nighttime and daytime discharged patients (Table 1). The frequency of weekend ICU discharge did not differ between nighttime and daytime ICU discharged patients [19.0% vs. 20.1%; respectively, p = 0.557) (Table 1). Demographic characteristics of study participants after propensity score matching according to destination at index ICU discharge are shown in S3 Table.

**Table 1:** Baseline characteristics of study participants after propensity score matching.

Characteristics	All Patients 1934 (100.0%)	Nighttime 649 (33.6%)	Daytime 1285 (66.4%)	P value
Age, years (median, IQR)*	66 (51–79)	65 (52–79)	66 (51–79)	0.679^a^
Men, n (%)*	1114 (57.6)	369 (56.9)	745 (58.0)	0.638^b^
SAPS III score (median, IQR)^§^*	43 (33–55)	42 (33–55)	44 (33–55)	0.696^a^
Reason for index ICU admission, n (%)*				0.714^b^
Medical	1200 (62.0)	399 (61.5)	801 (62.3)	
Surgical	734 (38.0)	250 (38.5)	484 (37.7)	
Admission source, n (%)*				0.970^b^
Operating room/procedure unit	715 (37.0)	244 (37.6)	471 (36.7)	
Emergency department	792 (41.0)	265 (40.8)	527 (41.0)	
Ward	223 (11.5)	71 (10.9)	152 (11.8)	
Step down unit	104 (5.4)	34 (5.2)	70 (5.4)	
Others^ǂ^	100 (5.2)	35 (5.4)	65 (5.1)	
Underlying disease, n (%)				
Systemic hypertension*	1037 (53.6)	338 (52.1)	699 (54.4)	0.335^b^
Diabetes mellitus*	589 (30.5)	194 (29.9)	395 (30.7)	0.702^b^
Cancer*	411 (21.3)	144 (22.2)	267 (20.8)	0.474^b^
Congestive heart failure*	240 (12.4)	79 (12.2)	161 (12.5)	0.822^b^
COPD*	166 (8.6)	58 (8.9)	108 (8.4)	0.693^b^
Chronic kidney disease requiring long-term dialysis	154 (8.0)	53 (8.2)	101 (7.9)	0.814^b^
Chronic kidney disease*	113 (5.8)	35 (5.4)	78 (6.1)	0.549^b^
Liver cirrhosis*	101 (5.2)	35 (5.4)	66 (5.1)	0.811^b^
Nonoperative admission diagnoses, n (%)				0.129^b^
Sepsis	546 (45.5)	190 (47.6)	356 (44.4)	
Cardiovascular	196 (16.3)	72 (18.0)	124 (15.5)	
Neurologic	137 (11.4)	30 (7.5)	107 (13.4)	
Respiratory	105 (8.8)	37 (9.3)	68 (8.5)	
Gastrointestinal	82 (6.8)	22 (5.5)	60 (7.5)	
Trauma	37 (3.1)	15 (3.8)	22 (2.7)	
Metabolic	43 (3.6)	16 (4.0)	27 (3.4)	
Other medical diseases	34 (2.8)	11 (2.8)	23 (2.9)	
Renal diseases	13 (1.1)	5 (1.3)	8 (1.0)	
Hematologic	7 (0.6)	1 (0.3)	6 (0.7)	
Operative admission diagnoses, n (%)				0.457^b^
Cardiovascular	206 (28.1)	67 (26.8)	139 (28.7)	
Gastrointestinal	157 (21.4)	52 (20.8)	105 (21.7)	
Orthopedic	134 (18.3)	42 (16.8)	92 (19.0)	
Renal	92 (12.5)	36 (14.4)	56 (11.6)	
Neurologic	71 (9.7)	24 (9.6)	47 (9.7)	
Respiratory	57 (7.8)	20 (8.0)	37 (7.6)	
Gynecologic	15 (2.0)	7 (2.8)	8 (1.7)	
Trauma	2 (0.3)	2 (0.8)	0 (0.0)	
Support at ICU admission, n (%)				
Vasopressors	284 (14.7)	84 (12.9)	200 (15.6)	0.124^b^
Mechanical ventilation	279 (14.4)	87 (13.4)	192 (14.9)	0.364^b^
Noninvasive ventilation	165 (8.5)	60 (9.2)	105 (8.2)	0.425^b^
Renal replacement therapy	7 (0.4)	4 (0.6)	3 (0.2)	0.186^b^
Support during index ICU stay, n (%)				
Vasopressors*	514 (26.6)	171 (26.3)	343 (26.7)	0.871^b^
Mechanical ventilation*	400 (27.7)	135 (20.8)	265 (20.6)	0.927^b^
Noninvasive ventilation*	462 (23.9)	156 (24.0)	306 (23.8)	0.913^b^
Renal replacement therapy*	190 (9.8)	66 (10.2)	124 (9.6)	0.717^b^
Destination at index ICU discharge, n (%)*				0.858^b^
Step-down unit	1184 (61.2)	401 (61.8)	783 (60.9)	
Ward	629 (32.5)	206 (31.7)	423 (32.9)	
Other/unknown^ǂ^	121 (6.3)	42 (6.5)	79 (6.1)	
Weekend ICU discharge, n (%)*	381 (19.7)	123 (19.0)	258 (20.1)	0.557^b^

Values represent median (IQR) or n (%). P values were calculated with the use of (a) Mann-Whitney U test or (b) chi-square test.

* Patients characteristics included into propensity score matching, SAPS III: simplified acute physiology score III,

^§^: scores on SAPS III range from 0 to 217, with higher scores indicating more severe illness and higher risk of death, COPD: chronic obstructive pulmonary disease,

^ǂ^: another hospital and home care,

^#^: home, another hospital, another ICU, hospice and home care.

#### Outcomes

In-hospital mortality was 6.5% (42/649 patients) in nighttime discharged patients compared to 5.6% (72/1,285 patients) daytime discharged patients (OR, 1.17; 95% CI, 0.79 to 1.73; p = 0.444) (Table 2). Frequency of ICU readmission was 10.5% (68/649 patients) in nighttime and 11.0% (141/1,285 patients) in daytime ICU discharge patients (OR, 0.95; 95% CI, 0.78 to 1.29; p = 0.741) (Table 2). The length of ICU stay [median (IQR)] was slightly lower in nighttime [1 (1–3) days] compared to daytime [2 (1–3) days] ICU discharged patients (p = 0.047) while the length of hospital stay did not differ between the groups (Table 2).

**Table 2:** Outcomes after propensity score matching.

Characteristics	All Patients 1934 (100.0%)	Nighttime 649 (33.6%)	Daytime 1285 (66.4%)	OR (95%CI)	P value
In-hospital mortality, n (%)	114 (5.9)	42 (6.5)	72 (5.6)	1.17 (0.79 to 1.73)	0.444^a^
ICU readmission, n (%)	209 (10.8)	68 (10.5)	141 (11.0)	0.95 (0.78 to 1.29)	0.741^a^
Number of ICU readmissions, median (IQR)	1 (1–1)	1 (1–1)	1 (1–2)		0.212^b^
Length of ICU stay (days), median (IQR)*	2 (1–3)	1 (1–3)	2 (1–3)		0.047^b^
Length of hospital stay (days), median (IQR)	9 (5–20)	10 (5–21)	9 (5–19)		0.440^b^

Values represent median (IQR) or n (%). OR: odds ratio, CI: confidence interval. P values were calculated with the use of (a) chi-square test and or (b) Mann-Whitney U test.

* Patients characteristics included into propensity score matching,

#### Outcomes according to location after ICU discharge

In-hospital mortality of patients discharged to the ward was 2.9% (6/206 patients) in nighttime compared to 5.0% (21/423 patients) in daytime ICU discharged patients (OR, 0.57; 95% CI, 0.23 to 1.45; p = 0.233) (Table 3). In-hospital mortality of ICU discharged patients to the step-down unit was 8.0% (32/401 patients) in nighttime compared to 6.3% (49/783 patients) in daytime discharged patients (OR, 1.30; 95% CI, 0.82 to 2.06; p = 0.267) (Table 3). The frequency of ICU readmission, length of ICU and hospital stay did not differ between nighttime and daytime ICU discharged patients to the ward or to the step-down unit (Table 3).

**Table 3:** Outcomes according to time and destination of ICU discharge.

Characteristics	All Patients	Nighttime	Daytime	P value
**Unit destination: Ward, n (%)**	**629 (100.0%)**	**206 (32.8%)**	**423 (67.2%)**	
In-hospital mortality, n (%)	27 (4.3)	6 (2.9)	21 (5.0)	0.233^a^
ICU readmission, n (%)	67 (10.7)	20 (9.7)	47 (11.1)	0.593^a^
Number of ICU readmissions, median (IQR)	1 (1–2)	1 (1–2)	1 (1–2)	0.688^b^
Weekend ICU discharge, n (%)	134 (21.3)	41 (19.9)	93 (22.0)	0.549^a^
Length of ICU stay (days), median (IQR)	1 (1–2)	1 (1–2)	1 (1–2)	0.051^b^
Length of hospital stay (days), median (IQR)	7 (4–14)	8 (4–15)	7 (4–13)	0.986^b^
**Unit destination: Step-down unit, n (%)**	**1,184 (100.0%)**	**401 (33.9%)**	**783 (66.1%)**	
In-hospital mortality, n (%)	81 (6.8)	32 (8.0)	49 (6.3)	0.267^a^
ICU readmission, n (%)	135 (11.4)	44 (11.0)	91 (11.6)	0.739^a^
Number of ICU readmissions, median (IQR)	1 (1–1)	1 (1–1)	1 (1–1)	0.460^b^
Weekend ICU discharge, n (%)	220 (18.6)	74 (18.5)	146 (18.6)	0.936^a^
Length of ICU stay (days), median (IQR)	2 (1–4)	2 (1–4)	2 (1–4)	0.156^b^
Length of hospital stay (days), median (IQR)	11 (6–24)	11 (6–26)	11 (6–22)	0.502^b^

Values represent median (IQR) or n (%). P values were calculated with the use of (a) chi-square test or (b) Mann-Whitney U test.

## Discussion

The main finding of this single center propensity-matched retrospective cohort study was that time of ICU discharge did not affect in-hospital mortality nor the frequency of ICU readmissions. Additionally, discharge facility, i.e., wards or step-down unit, did not affect in-hospital mortality nor incidence of ICU readmissions.

In agreement with our results, Uusaro et al. reported in a large multicenter retrospective cohort study involving 18 ICUs and 20,623 ICU discharges in Finland that “out-of-office” hours ICU discharge, defined as those occurring from 4:00 pm to 8:00 am, was not associated with increased in-hospital mortality (adjusted OR, 0.98; 95% CI, 0.85 to 1.13) [31]. In another retrospective cohort study with three ICUs at Mayo Medical Center, USA, in which only 3.6% of ICU discharges occurred during nighttime, i.e., between 7:00 pm and 6:59 am, time of ICU discharge was not associated with increased in-hospital mortality (OR, 1.04; 95% CI, 0.64 to 1.70; p = 0.860) [11]. Nevertheless, nighttime ICU discharge was associated with an increased rate of ICU readmission when compared to daytime ICU discharge (12.2% vs. 9.0%, respectively, p = 0.027) [11]. Finally, in a large prospective, multicenter observational study involving 40 ICUs in Australia and New Zealand, time of discharge was also not associated with increased hospital mortality [32].

In contrast, most of the previously published studies have shown that patients discharged from the ICU to the wards at night are at a higher risk of dying in the hospital compared with patients discharged during the day [6, 10, 16, 17, 21, 27, 33–37]. Nevertheless, Beck and cols. demonstrated in a retrospective cohort study including nine ICUs in the UK that discharge time was not associated with increased hospital mortality when patients are discharged to a step-down unit [21]. In our study, approximately 60% of patients were discharged to a step-down unit, which may have contributed to the lack of association between time of transfer and worse outcomes.

The frequency of nighttime ICU discharge reported in literature can vary widely, ranging from 3.6% [11] up to 35% [36]. Goldfrad et al. reported in a multicenter retrospective cohort study with 88 ICUs in the UK a two-fold increase in the frequency of nighttime (10:00 pm to 6:59 am) ICU discharges between 1988–1990 and 1995–1998 [6]. Moreover, in this study, premature ICU discharge, i.e., early ICU discharge due to shortage of ICU beds, was more common at night than during the day (42.6% vs. 5.0%, respectively) [6]. In our study, 15.1% of patients (650/4,313 patients) were discharged from the ICU during nighttime and the ICU and hospital occupancy rate usually remained below 85%. We can only speculate that the reason for nighttime ICU discharge in our study does not lie in the number of beds, but rather in the inability of the ward to accept patients discharged from the ICU in a timely manner. Furthermore, the absence of ICU bed supply constraint and, conversely, low prevalence of premature ICU discharge, may explain the observed lack of association between time of ICU discharge and poor outcomes.

Our study has limitations. This was a single center study carried out in a large private tertiary-care metropolitan teaching hospital located in São Paulo. Thus, our findings may neither be generalized to other ICUs in Brazil nor to other developing countries where healthcare systems, patient’s severity of illness both at ICU admission and discharge, policies for ICU admission and discharge, surveillance and destination after ICU discharge, and pressure for ICU beds may differ considerably from our center despite the fact we included a broad range of medical and surgical patients. Secondly, we used a propensity score design aiming to mitigate confounding and enhance internal validity of this analysis. Nevertheless, although a propensity score design helped to account for inherent differences in patient characteristics between the groups, we cannot guarantee that it mitigates confounding completely [28]. Finally, we did not address organ dysfunction at the time of ICU discharge. It has been shown that residual organ dysfunction within 24 hours before ICU discharge is associated with decreased long-term survival [38]. Therefore, residual organ dysfunction at the time of ICU discharge might have affected decision making by intensivists regarding the time of discharge and destination unit.

## Conclusion

In this propensity-matched single center retrospective cohort study performed in an environment with no demand pressure for ICU beds, time of ICU discharge did not affect in-hospital mortality nor the frequency of ICU readmissions. Further large-scale multicenter prospective studies are needed to improve our understanding of the relationship between time of ICU discharge and outcomes.

## Supporting information

S1 Fig Distribution of propensity scores along with kernel density estimates in nighttime and daytime ICU discharged groups before and after matching. Treated: represents group nighttime ICU discharged and control represents daytime ICU discharged group.
(DOCX)

S2 Fig Absolute standardized differences comparing baseline covariates between nighttime and daytime discharged patients in the unmatched and matched cohorts. White open circles represent unmatched (Before propensity score matching) and black filled circles matched (after propensity score matching) cohorts. The nineteen clinically relevant patients characteristics were entered in a logistic regression for propensity score estimation as follows: age (years), gender (0 = female, 1 = male), SAPS III score (points), reason for index ICU admission (0 = surgical, 1 = medical), index admission source (0 = emergency department, 1 = ward, 2 = step down unit, 3 = operating room/procedure, 4 = others), presence of systemic hypertension (0 = no, 1 = yes), diabetes mellitus (0 = no, 1 = yes), cancer (0 = no, 1 = yes), congestive heart failure (0 = no, 1 = yes), COPD = chronic obstructive pulmonary disease (0 = no, 1 = yes), chronic kidney disease (0 = no, 1 = yes) and liver cirrhosis (0 = no, 1 = yes), supportive therapy during the ICU stay [need for vasopressors (0 = no, 1 = yes), mechanical ventilation (0 = no, 1 = yes), NIV = noninvasive mechanical ventilation (0 = no, 1 = yes) and RRT = renal replacement therapy (0 = no, 1 = yes)], index weekend ICU discharge (0 = no, 1 = yes), ICU Length of ICU stay (days) and destination at index ICU discharge (0 = ward, 1 = step down unit, 3 = other/unknown).
(DOCX)

S1 Table Baseline characteristics of study participants before propensity score matching. Values represent median (IQR) or n (%). SAPS III: simplified acute physiology score III, §: scores on SAPS III range from 0 to 217, with higher scores indicating more severe illness and higher risk of death, COPD: chronic obstructive pulmonary disease, ǂ: another hospital and home care, #: home, another hospital, another ICU, hospice and home care. P values were calculated with the use of (a) Mann-Whitney U test and (b) chi-square test.
(DOCX)

S2 Table Outcomes before propensity score matching. Values represent median (IQR) or n (%). OR: odds ratio, CI: confidence interval. P values were calculated with the use of a (a) chi-square test and or (b) Mann-Whitney U test.
(DOCX)

S3 Table Characteristics of study participants accordingly to the destination at index ICU discharge. Values represent median (IQR) or n (%). SAPS III: simplified acute physiology score III, §: scores on SAPS III range from 0 to 217, with higher scores indicating more severe illness and higher risk of death, COPD: chronic obstructive pulmonary disease, ǂ: another hospital and home care, P values were calculated with the use of (a) Mann-Whitney U test or (b) chi-square test.
(DOCX)

## Abbreviations

CI: confidence interval
ICU: Intensive care unit
IQR: interquartile ranges
LOS: length of stay
OR: odds ratio
SAPS 3 score: Simplified Acute Physiology score
