# Supplementary material for: Nighttime intensive care unit discharge and outcomes: A propensity matched retrospective cohort study
Source: PLoS One. 2018 Dec 13;13(12):e0207268. doi: 10.1371/journal.pone.0207268 (PMC6292615; doi:10.1371/journal.pone.0207268)
Supplement: S1 Fig — Treated: represents group nighttime ICU discharged and control represents daytime ICU discharged group. (DOCX) [file pone.0207268.s001.docx]

**S1 Fig.** Distribution of propensity scores along with kernel density estimates in nighttime and daytime ICU discharged groups before and after matching.


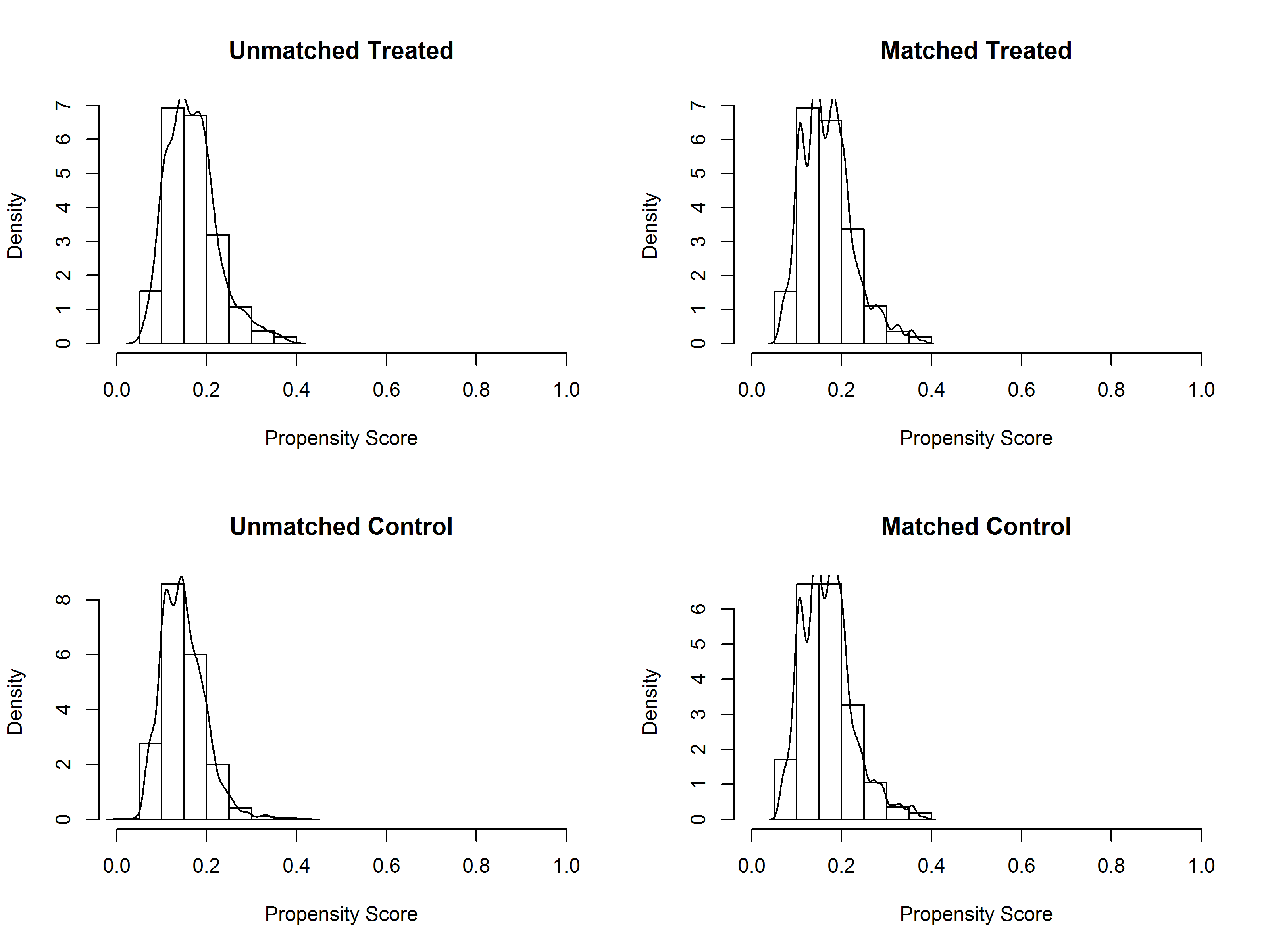


Treated: represents group nighttime ICU discharged and control represents daytime ICU discharged group.
