# Supplementary material for: Nighttime intensive care unit discharge and outcomes: A propensity matched retrospective cohort study
Source: PLoS One. 2018 Dec 13;13(12):e0207268. doi: 10.1371/journal.pone.0207268 (PMC6292615; doi:10.1371/journal.pone.0207268)
Supplement: S2 Fig — White open circles represent unmatched (Before propensity score matching) and black filled circles matched (after propensity score matching) cohorts. The nineteen clinically relevant patients characteristics were entered in a logistic regression for propensity score estimation as follows: age (years), gender (0 = female, 1 = male), SAPS III score (points), reason for index ICU admission (0 = surgical, 1 = medical), index admission source (0 = emergency department, 1 = ward, 2 = step down unit, 3 = operating room/procedure, 4 = others), presence of systemic hypertension (0 = no, 1 = yes), diabetes mellitus (0 = no, 1 = yes), cancer (0 = no, 1 = yes), congestive heart failure (0 = no, 1 = yes), COPD = chronic obstructive pulmonary disease (0 = no, 1 = yes), chronic kidney disease (0 = no, 1 = yes) and liver cirrhosis (0 = no, 1 = yes), supportive therapy during the ICU stay [need for vasopressors (0 = no, 1 = yes), mechanical ventilation (0 = no, 1 = yes), NIV = noninvasive mechanical ventilation (0 = no, 1 = yes) and RRT = renal replacement therapy (0 = no, 1 = yes)], index weekend ICU discharge (0 = no, 1 = yes), ICU Length of ICU stay (days) and destination at index ICU discharge (0 = ward, 1 = step down unit, 3 = other/unknown). (DOCX) [file pone.0207268.s002.docx]

**S2 Fig.** Absolute standardized differences comparing baseline covariates between nighttime and daytime discharged patients in the unmatched and matched cohorts.


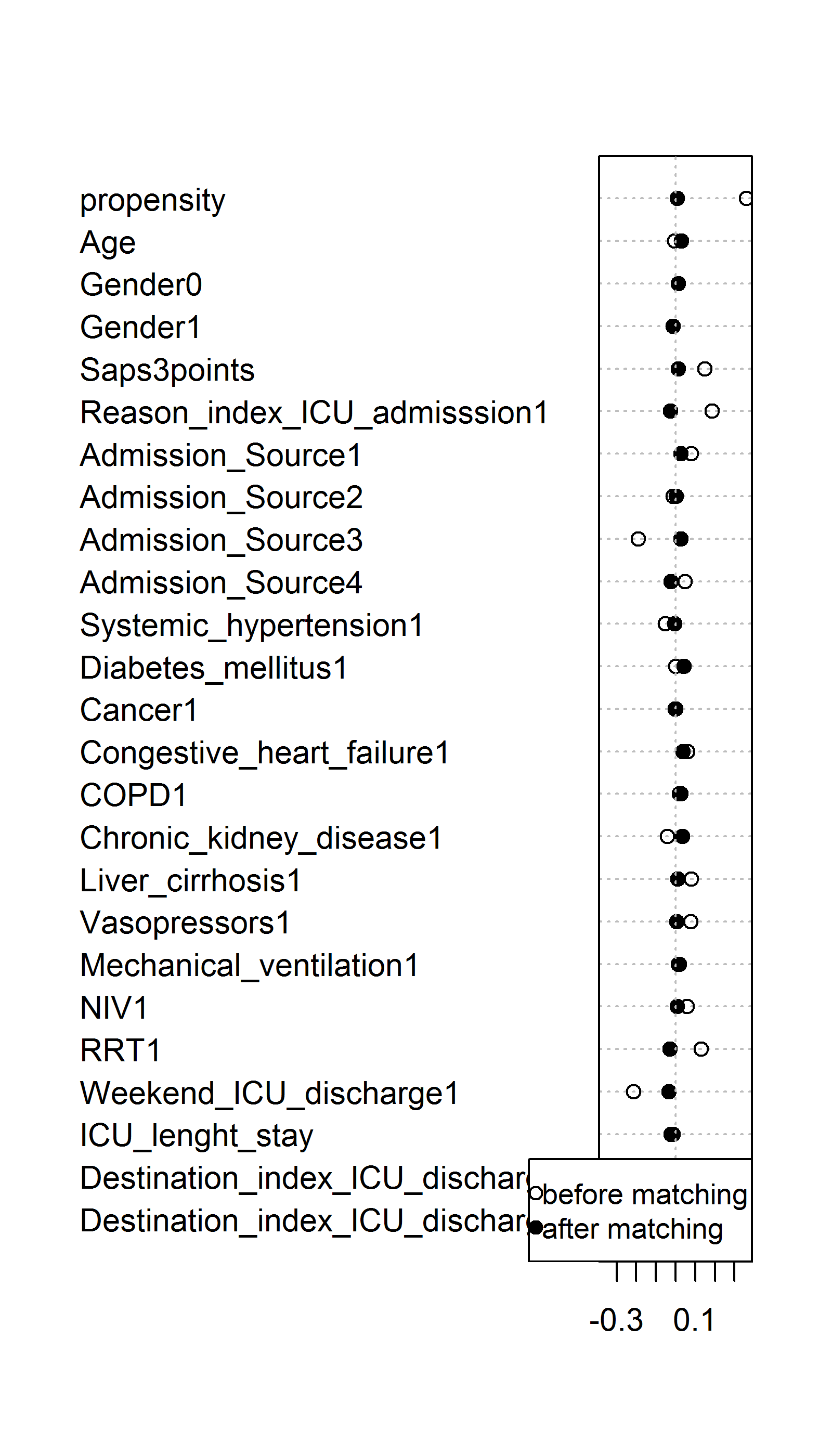


White open circles represent unmatched (Before propensity score matching) and black filled circles matched (after propensity score matching) cohorts. The nineteen clinically relevant patients characteristics were entered in a logistic regression for propensity score estimation as follows: age (years), gender (0=female, 1=male), SAPS III score (points), reason for index ICU admission (0=surgical, 1=medical), index admission source (0=emergency department, 1=ward, 2=step down unit, 3=operating room/procedure, 4=others), presence of systemic hypertension (0=no, 1=yes), diabetes mellitus (0=no, 1=yes), cancer (0=no, 1=yes), congestive heart failure (0=no, 1=yes), COPD =chronic obstructive pulmonary disease (0=no, 1=yes), chronic kidney disease (0=no, 1=yes) and liver cirrhosis (0=no, 1=yes), supportive therapy during the ICU stay [need for vasopressors (0=no, 1=yes), mechanical ventilation (0=no, 1=yes), NIV=noninvasive mechanical ventilation (0=no, 1=yes) and RRT=renal replacement therapy (0=no, 1=yes)], index weekend ICU discharge (0=no, 1=yes), ICU Length of ICU stay (days) and destination at index ICU discharge (0=ward, 1=step down unit, 3=other/unknown).
