# Supplementary material for: Nighttime intensive care unit discharge and outcomes: A propensity matched retrospective cohort study
Source: PLoS One. 2018 Dec 13;13(12):e0207268. doi: 10.1371/journal.pone.0207268 (PMC6292615; doi:10.1371/journal.pone.0207268)
Supplement: S1 Table — Values represent median (IQR) or n (%). SAPS III: simplified acute physiology score III, §: scores on SAPS III range from 0 to 217, with higher scores indicating more severe illness and higher risk of death, COPD: chronic obstructive pulmonary disease, ǂ: another hospital and home care, #: home, another hospital, another ICU, hospice and home care. P values were calculated with the use of (a) Mann-Whitney U test and (b) chi-square test. (DOCX) [file pone.0207268.s003.docx]

**S1 Table. Baseline characteristics of study participants before propensity score matching.**

| **Characteristics** | **All Patients**  **4,313 (100.0%)** | **Nighttime**  **650 (15.1%)** | **Daytime**  **3,663 (84.9%)** | **P value** |
| --- | --- | --- | --- | --- |
| Age, years (median, IQR) | 65 (51-79) | 65 (52-79) | 66 (51-79) | 0.822^a^ |
| Men, n (%) | 2471 (57.3) | 369 (56.8) | 2102 (57.4) | 0.770^b^ |
| SAPS III score (median, IQR)^§^ | 41 (31-53) | 42 (33-55) | 40 (31-52) | <0.001^a^ |
| Reason for index ICU admission, n (%) |  |  |  | <0.001^b^ |
| Medical | 2318 (53.7) | 399 (61.4) | 1919 (52.4) |  |
| Surgical | 1995 (46.3) | 251 (38.6) | 1744 (47.6) |  |
| Admission source, n (%) |  |  |  | <0.001^b^ |
| Operating room/procedure unit | 1962 (45.5) | 245 (37.7) | 1717 (46.9) |  |
| Emergency department | 1543 (35.8) | 265 (40.8) | 1278 (34.9) |  |
| Ward | 379 (8.8) | 71 (10.9) | 308 (8.4) |  |
| Step down unit | 236 (5.5) | 34 (5.2) | 202 (5.5) |  |
| Others^ǂ^ | 193 (4.5) | 35 (5.4) | 158 (4.3) |  |
| Underlying disease, n (%) |  |  |  |  |
| Systemic hypertension | 2341 (54.3) | 338 (52.0) | 2003 (54.7) | 0.206^b^ |
| Diabetes mellitus | 1287 (29.8) | 194 (29.8) | 1093 (29.8) | 0.997^b^ |
| Cancer | 956 (22.2) | 144 (22.2) | 812 (22.2) | 0.994^b^ |
| Congestive heart failure | 450 (10.4) | 79 (12.2) | 371 (10.1) | 0.120^b^ |
| COPD | 358 (8.3) | 58 (8.9) | 300 (8.2) | 0.532^b^ |
| Chronic kidney disease requiring  long-term dialysis | 319 (7.4) | 53 (8.2) | 266 (7.3) | 0.423^b^ |
| Chronic kidney disease | 267 (6.2) | 35 (5.4) | 232 (6.3) | 0.355^b^ |
| Liver cirrhosis | 172 (4.0) | 36 (5.5) | 136 (3.7) | 0.028^b^ |
| Nonoperative admission diagnoses, n (%) |  |  |  | 0.101^b^ |
| Sepsis | 1034 (44.6) | 190 (47.6) | 844 (44.0) |  |
| Cardiovascular | 384 (16.6) | 72 (18.0) | 312 (16.3) |  |
| Neurologic | 276 (11.9) | 30 (7.5) | 246 (12.8) |  |
| Respiratory | 216 (9.3) | 37 (9.3) | 179 (9.3) |  |
| Gastrointestinal | 166 (7.2) | 22 (5.5) | 144 (7.5) |  |
| Trauma | 77 (3.3) | 15 (3.8) | 62 (3.2) |  |
| Metabolic | 73 (3.1) | 16 (4.0) | 57 (3.0) |  |
| Other medical diseases | 60 (2.6) | 11 (2.8) | 49 (2.6) |  |
| Renal diseases | 19 (0.8) | 5 (1.3) | 14 (0.7) |  |
| Hematologic | 13 (0.6) | 1 (0.3) | 12 (0.6) |  |
| Operative admission diagnoses, n (%) |  |  |  | 0.236^b^ |
| Cardiovascular | 530 (26.6) | 67 (26.7) | 463 (26.5) |  |
| Gastrointestinal | 441 (22.1) | 53 (21.1) | 388 (22.2) |  |
| Orthopedic | 339 (17.0) | 42 (16.7) | 297 (17.0) |  |
| Renal | 263 (13.2) | 36 (14.3) | 227 (13.0) |  |
| Neurologic | 201 (10.1) | 24 (9.6) | 177 (10.1) |  |
| Respiratory | 175 (8.8) | 20 (8.0) | 155 (8.9) |  |
| Gynecologic | 43 (2.2) | 7 (2.8) | 36 (2.1) |  |
| Trauma | 3 (0.2) | 2 (0.8) | 1 (0.1) |  |
| Support at ICU admission, n (%) |  |  |  |  |
| Vasopressors | 566 (13.1) | 85 (13.1) | 481 (13.1) | 0.970^b^ |
| Mechanical ventilation | 643 (14.9) | 88 (13.5) | 555 (15.2) | 0.287^b^ |
| Noninvasive ventilation | 324 (7.5) | 60 (9.2) | 264 (7.2) | 0.071^b^ |
| Renal replacement therapy | 12 (0.3) | 4 (0.6) | 8 (0.2) | 0.077^b^ |
| Support during index ICU stay, n (%) |  |  |  |  |
| Vasopressors | 1016 (23.6) | 172 (26.5) | 844 (23.0) | 0.058^b^ |
| Mechanical ventilation | 881 (20.4) | 136 (20.9) | 745 (20.3) | 0.733^b^ |
| Noninvasive ventilation | 944 (21.9) | 156 (24.0) | 788 (21.5) | 0.157^b^ |
| Renal replacement therapy | 301 (7.0) | 67 (10.3) | 234 (6.4) | <0.001^b^ |
| Destination at index ICU discharge, n (%) |  |  |  | 0.001^b^ |
| Step-down unit | 2493 (57.8) | 402 (61.8) | 2091 (57.1) |  |
| Ward | 1618 (37.5) | 206 (31.7) | 1412 (38.5) |  |
| Other/unknown^#^ | 202 (4.7) | 42 (6.5) | 160 (4.4) |  |
| Weekend ICU discharge, n (%) | 1123 (26.0) | 123 (18.9) | 1000 (27.3) | <0.001^b^ |

Values represent median (IQR) or n (%). SAPS III: simplified acute physiology score III, §: scores on SAPS III range from 0 to 217, with higher scores indicating more severe illness and higher risk of death, COPD: chronic obstructive pulmonary disease, ǂ: another hospital and home care, #: home, another hospital, another ICU, hospice and home care. P values were calculated with the use of (a) Mann-Whitney U test and (b) chi-square test.
