# Supplementary material for: Nighttime intensive care unit discharge and outcomes: A propensity matched retrospective cohort study
Source: PLoS One. 2018 Dec 13;13(12):e0207268. doi: 10.1371/journal.pone.0207268 (PMC6292615; doi:10.1371/journal.pone.0207268)
Supplement: S2 Table — Values represent median (IQR) or n (%). OR: odds ratio, CI: confidence interval. P values were calculated with the use of a (a) chi-square test and or (b) Mann-Whitney U test. (DOCX) [file pone.0207268.s004.docx]

**S2 Table. Outcomes before propensity score matching.**

| **Characteristics** | **All Patients**  **4,313 (100.0%)** | **Nighttime**  **650 (15.1%)** | **Daytime**  **3,663 (84.9%)** | **OR (95%CI)** | **P value** |
| --- | --- | --- | --- | --- | --- |
| In-hospital mortality, n (%) | 229 (5.3) | 42 (6.5) | 187 (5.1) | 1.28 (0.91 to 1.81) | 0.156^a^ |
| ICU readmission, n (%) | 431 (10.0) | 68 (10.5) | 363 (9.9) | 1.06 (0.81 to 1.40) | 0.666^a^ |
| Number of ICU readmissions, median (IQR) | 1 (1-1) | 1 (1-1) | 1 (1-1) |  | 0.379^b^ |
| Length of ICU stay (days), median (IQR) | 2 (1-3) | 1 (1-3) | 2 (1-3) |  | 0.166^b^ |
| Length of hospital stay (days), median (IQR) | 9 (5-18) | 10 (5-21) | 9 (5-18) |  | 0.150^b^ |

Values represent median (IQR) or n (%). OR: odds ratio, CI: confidence interval. P values were calculated with the use of a (a) chi-square test and or (b) Mann-Whitney U test.
