# Supplementary material for: Nighttime intensive care unit discharge and outcomes: A propensity matched retrospective cohort study
Source: PLoS One. 2018 Dec 13;13(12):e0207268. doi: 10.1371/journal.pone.0207268 (PMC6292615; doi:10.1371/journal.pone.0207268)
Supplement: S3 Table — Values represent median (IQR) or n (%). SAPS III: simplified acute physiology score III, §: scores on SAPS III range from 0 to 217, with higher scores indicating more severe illness and higher risk of death, COPD: chronic obstructive pulmonary disease, ǂ: another hospital and home care, P values were calculated with the use of (a) Mann-Whitney U test or (b) chi-square test. (DOCX) [file pone.0207268.s005.docx]

**S3 Table. Characteristics of study participants accordingly to the destination at index ICU discharge.**

| **Location after ICU discharge** | **Ward** | | |  | **Step-down unit** | | |  |
| --- | --- | --- | --- | --- | --- | --- | --- | --- |
| **Characteristics** | **All Patients**  **629 (100.0%)** | **Nighttime**  **206 (32.8%)** | **Daytime**  **423 (67.2%)** | **P value** | **All Patients**  **1,184 (100.0%)** | **Nighttime**  **401 (33.9%)** | **Daytime**  **783 (66.1%)** | **P value** |
| Age, years (median, IQR) | 58 (47-70) | 59 (48-72) | 58 (46-68) | 0.115^a^ | 71 (55-82) | 69 (54-82) | 72 (56-83) | 0.063^a^ |
| Men, n (%) | 361 (57.4) | 108 (52.4) | 253 (59.8) | 0.079^b^ | 690 (58.3) | 240 (59.9) | 450 (57.5) | 0.432^b^ |
| SAPS III score (median, IQR)^§^ | 38 (28-48) | 38 (27-48) | 37 (28-50) | 0.713^a^ | 47 (36-57) | 47 (35-57) | 47 (37-57) | 0.291^a^ |
| Reason for index ICU admission, n (%) |  |  |  | 0.434^b^ |  |  |  | 0.717^b^ |
| Medical | 298 (47.4) | 93 (45.1) | 205 (48.5) |  | 820 (69.3) | 275 (68.6) | 545 (69.6) |  |
| Surgical | 331 (52.6) | 113 (54.9) | 218 (51.5) |  | 364 (30.7) | 126 (31.4) | 238 (30.4) |  |
| Admission source, n (%) |  |  |  | 0.870^b^ |  |  |  | 0.798^b^ |
| Operating room/procedure unit | 323 (51.4) | 110 (53.4) | 213 (50.4) |  | 356 (30.1) | 125 (31.2) | 231 (29.5) |  |
| Emergency department | 186 (29.6) | 57 (27.7) | 129 (30.5) |  | 551 (46.5) | 186 (46.4) | 365 (46.6) |  |
| Ward | 90 (14.3) | 31 (15.0) | 59 (13.9) |  | 116 (9.8) | 34 (8.5) | 82 (10.5) |  |
| Step down unit | 11 (1.7) | 3 (1.5) | 8 (1.9) |  | 85 (7.2) | 28 (7.0) | 57 (7.3) |  |
| Others^ǂ^ | 19 (3.0) | 5 (2.4) | 14 (3.3) |  | 76 (6.4) | 28 (7.0) | 48 (6.1) |  |
| Underlying disease, n (%) |  |  |  |  |  |  |  |  |
| Systemic hypertension | 287 (45.6) | 93 (45.1) | 194 (45.9) | 0.865^b^ | 686 (57.9) | 227 (56.6) | 459 (58.6) | 0.507^b^ |
| Diabetes mellitus | 154 (24.5) | 47 (22.8) | 107 (25.3) | 0.497^b^ | 396 (33.4) | 133 (33.2) | 263 (33.6) | 0.884^b^ |
| Cancer | 114 (18.1) | 44 (21.4) | 70 (16.5) | 0.142^b^ | 276 (23.3) | 94 (23.4) | 182 (23.2) | 0.939^b^ |
| Congestive heart failure | 27 (4.3) | 10 (4.9) | 17 (4.0) | 0.628^b^ | 196 (16.6) | 64 (16.0) | 132 (16.9) | 0.694^b^ |
| COPD | 31 (4.9) | 14 (6.8) | 17 (4.0) | 0.131^b^ | 130 (11.0) | 43 (10.7) | 87 (11.1) | 0.840^b^ |
| Chronic kidney disease requiring  long-term dialysis | 86 (13.7) | 33 (16.0) | 53 (12.5) | 0.232^b^ | 57 (4.8) | 17 (4.2) | 40 (5.1) | 0.508^b^ |
| Chronic kidney disease | 25 (4.0) | 5 (2.4) | 20 (4.7) | 0.166^b^ | 85 (7.2) | 28 (7.0) | 57 (7.3) | 0.851^b^ |
| Liver cirrhosis | 71 (11.3) | 20 (9.7) | 51 (12.1) | 0.382^b^ | 24 (2.0) | 11 (2.7) | 13 (1.7) | 0.211^b^ |
| Nonoperative admission diagnoses, n (%) |  |  |  | 0.061^b^ |  |  |  | 0.555^b^ |
| Sepsis | 141 (47.3) | 45 (48.4) | 96 (46.8) |  | 377 (46.0) | 134 (48.7) | 243 (44.6) |  |
| Cardiovascular | 36 (12.1) | 15 (16.1) | 21 (10.2) |  | 143 (17.4) | 53 (19.3) | 90 (16.5) |  |
| Neurologic | 16 (5.4) | 0 (0.0) | 16 (7.8) |  | 113 (13.8) | 28 (10.2) | 85 (15.6) |  |
| Respiratory | 30 (10.1) | 12 (12.9) | 18 (8.8) |  | 68 (8.3) | 22 (8.0) | 46 (8.4) |  |
| Gastrointestinal | 37 (12.4) | 8 (8.6) | 29 (14.1) |  | 40 (4.9) | 11 (4.0) | 29 (5.3) |  |
| Trauma | 5 (1.7) | 1 (1.1) | 4 (2.0) |  | 26 (3.2) | 10 (3.6) | 16 (2.9) |  |
| Metabolic | 11 (3.7) | 5 (5.4) | 6 (2.9) |  | 24 (2.9) | 9 (3.3) | 15 (2.8) |  |
| Other medical diseases | 14 (4.7) | 6 (6.5) | 8 (3.9) |  | 18 (2.2) | 4 (1.5) | 14 (2.6) |  |
| Renal diseases | 3 (1.0) | 1 (1.1) | 2 (1.0) |  | 9 (1.1) | 3 (1.1) | 6 (1.1) |  |
| Hematologic | 5 (1.7) | 0 (0.0) | 5 (2.4) |  | 2 (0.2) | 1 (0.4) | 1 (0.2) |  |
| Operative admission diagnoses, n (%) |  |  |  | 0.224^b^ |  |  |  | 0.779^b^ |
| Cardiovascular | 48 (14.5) | 12 (10.6) | 36 (16.5) |  | 136 (37.4) | 50 (39.7) | 86 (36.1) |  |
| Gastrointestinal | 84 (25.4) | 25 (22.1) | 59 (27.1) |  | 66 (18.1) | 25 (19.8) | 41 (17.2) |  |
| Orthopedic | 73 (22.1) | 23 (20.4) | 50 (22.9) |  | 58 (15.9) | 18 (14.3) | 40 (16.8) |  |
| Renal | 74 (22.4) | 29 (25.7) | 45 (20.6) |  | 16 (4.4) | 6 (4.8) | 10 (4.2) |  |
| Neurologic | 24 (7.3) | 11 (9.7) | 13 (6.0) |  | 47 (12.9) | 13 (10.3) | 34 (14.3) |  |
| Respiratory | 20 (6.0) | 8 (7.1) | 12 (5.5) |  | 33 (9.1) | 11 (8.7) | 22 (9.2) |  |
| Gynecologic | 8 (2.4) | 5 (4.4) | 3 (1.4) |  | 7 (1.9) | 2 (1.6) | 5 (2.1) |  |
| Trauma | 0 (0.0) | 0 (0.0) | 0 (0.0) |  | 1 (0.3) | 1 (0.8) | 0 (0.0) |  |
| Support at ICU admission, n (%) |  |  |  |  |  |  |  |  |
| Vasopressors | 79 (12.6) | 26 (12.6) | 53 (12.5) | 0.974^b^ | 190 (16.0) | 52 (13.0) | 138 (17.6) | 0.039^b^ |
| Mechanical ventilation | 62 (9.9) | 22 (10.7) | 40 (9.5) | 0.629^b^ | 202 (17.1) | 56 (14.0) | 146 (18.6) | 0.043^b^ |
| Noninvasive ventilation | 35 (5.6) | 12 (5.8) | 23 (5.4) | 0.842^b^ | 121 (10.2) | 46 (11.5) | 75 (9.6) | 0.309^b^ |
| Renal replacement therapy | 4 (0.6) | 1 (0.5) | 3 (0.7) | 0.740^b^ | 2 (0.2) | 2 (0.5) | 0 (0.0) | 0.048^b^ |
| Support during index ICU stay, n (%) |  |  |  |  |  |  |  |  |
| Vasopressors | 144 (22.9) | 49 (23.8) | 95 (22.5) | 0.710^b^ | 344 (29.1) | 110 (27.4) | 234 (29.9) | 0.379^b^ |
| Mechanical ventilation | 88 (14.0) | 28 (13.6) | 60 (14.2) | 0.841^b^ | 289 (24.4) | 96 (23.9) | 193 (24.6) | 0.788^b^ |
| Noninvasive ventilation | 83 (13.2) | 24 (11.7) | 59 (13.9) | 0.424^b^ | 363 (30.7) | 125 (31.2) | 238 (30.4) | 0.784^b^ |
| Renal replacement therapy | 88 (14.0) | 26 (12.6) | 62 (14.7) | 0.490^b^ | 91 (7.7) | 35 (8.7) | 56 (7.2) | 0.335^b^ |

Values represent median (IQR) or n (%). SAPS III: simplified acute physiology score III, §: scores on SAPS III range from 0 to 217, with higher scores indicating more severe illness and higher risk of death, COPD: chronic obstructive pulmonary disease, ǂ: another hospital and home care, P values were calculated with the use of (a) Mann-Whitney U test or (b) chi-square test.
